# Strategies comparison in response to the two waves of COVID-19 in the United States and India

**DOI:** 10.1186/s12939-022-01666-9

**Published:** 2022-04-29

**Authors:** Junyan Yang, Leiyu Shi, Haiqian Chen, Xiaohan Wang, Jun Jiao, Manfei Yang, Meiheng Liu, Gang Sun

**Affiliations:** 1 Department of Health Management, School of Health Management, Southern Medical University, Guangzhou, 510515 Guangdong China; 2 Department of Health Policy and Management, Bloomberg School of Public Health, Johns Hopkins University, Baltimore, MD 21205 USA

**Keywords:** COVID-19, Non-pharmacotherapy intervention, The United States and India

## Abstract

**Background:**

This study aimed to compare the prevention and control strategies adopted by the United States and India in the COVID-19 outbreak and analyze the effectiveness of their strategies, in order to provide empirical experience for the prevention and control of the epidemic.

**Methods:**

This study extracted official data on COVID-19 from various official websites, summarized the policies in place in the United States and India, and evaluated the effectiveness of their policies.

**Results:**

The United States has adopted a series of mitigation strategies to control the two waves of epidemic, including strengthening virus detection, calling on the people to wear masks and so on. As of May 30, 2021, although the daily new cases there decreased to some extent, the effect was not ideal. The US’s daily new cases ranked fourth and the cumulative number of confirmed cases ranked first in the world. India has adopted containment strategies in the initial stage of the outbreak, making the epidemic relatively stable. In the later stage, India has turned to adopt mitigation strategies. In addition, many factors including the lack of medical resources and premature relaxation measures led to the rapid deterioration of the epidemic situation. As of May 30, 2021, although the daily new cases in India has a downward trend, it ranked first in the world, and the cumulative number of confirmed cases ranked second.

**Conclusion:**

There are differences between the epidemic prevention strategies adopted by the United States and India, especially India’s containment strategies which helped it better control the epidemic in the early stage. However, the epidemic in the two countries is still severe. With the advent of virus mutants and the absence of immune barriers, it is meaningful that the two countries continue to take non-pharmacotherapy intervention measures and accelerate vaccination, according to specific national conditions adopt containment strategies that can control the epidemic more quickly when necessary, and pay attention to the risk of epidemic rebound caused by premature relaxation of epidemic prevention policies.

## Background

In December 2019, an outbreak of a novel coronavirus SARS-CoV-2 was first reported in Wuhan, China [1]. On 11 March 2020, WHO Director-General declared that the COVID-19 outbreak has the characteristics of a global pandemic [2]. COVID-19 is the third major coronavirus outbreak after SARS-CoV and MERS-CoV, and has killed more people than any previous outbreak [3]. As of May 30, 2021, the cumulative number of confirmed COVID-19 cases worldwide has exceeded 170 million and the cumulative death toll has exceeded 3.54 million [4].

In response to COVID-19, governments around the world according to their actual situations adopted various interventions, such as calling for wearing masks, canceling or postponing large gatherings, increasing physical distance between patients infected with COVID-19 and uninfected people, conducting online teaching, and strengthening the screening, tracking and isolation of suspected and confirmed cases. In addition, in many countries, areas with serious epidemic situations have been blocked, entry control has been strict, round-trip flights with severely affected countries have been cut off, entry personnel have been strictly screened, and forced isolation has been carried out as required [5]. In terms of COVID-19 vaccines, many vaccines have been put into use, such as China's Sinovac COVID-19 vaccine, Sinopharm COVID-19 vaccine, Pfizer vaccine, AstraZeneca vaccine, etc [6]. Although more and more people are vaccinated globally, it is not advisable to rely on vaccines alone to fight the outbreak. Seychelles, as one of the countries with the largest number of vaccinations in the world, has doubled the number of confirmed cases by early May after lifting the restrictions on most tourists from March 25, 2021 [7]. Michael Z. Lin, associate professor of neurobiology and bioengineering at Stanford University, warned that what countries need to do was not only vaccination but also adequate border control, extensive testing, and the setting of hospitals capable of responding to the epidemic [7]. The study of Giulia Giordano et al. also shows that non-pharmacotherapy interventions have a greater impact on the evolution of the epidemic than vaccination alone [8]. In addition, in the face of the complex situation of virus mutation, the effect of vaccine may be affected to some extent. The official data released by the US CDC showed that the mRNA vaccine used in the United States was difficult to prevent the infection and transmission of Delta variant, although it has good efficacy in clinical trials [9]. It can be seen that non-pharmaceutical interventions still play a very important role in epidemic prevention and control at the present time when vaccination is accelerated.

At present, the top two countries in the world with the cumulative number of confirmed cases are the United States and India and the cumulative death toll of the United States ranks first and India ranks third in the world, which have attracted the world's attention. It is also very important for them to take appropriate non-pharmaceutical interventions. In addition, India began to implement the vaccination plan on January 16, 2021, but as of May 1, only nearly 10% of Indians have received one dose of vaccine and about 2% have completed two doses of vaccine [10]. The proportion of vaccination was very low. At the same time, the vaccination rate in the United States is far lower than the 70% needed to achieve herd immunity [11]. Therefore, it seems more necessary to take reasonable non-pharmaceutical intervention measures to alleviate the epidemic. Based on the background of the global COVID-19 epidemic, combined with the non-pharmaceutical intervention measures adopted by the United States and India and relevant official data, this article analyzed the implementation effects of the measures and brought some enlightenment for limiting the epidemic in the two countries and even the world.

## Material and methods

### Research setting

This study focused on the comparative analysis of non-pharmacotherapy interventions for COVID-19 in the United States and India from January 20, 2020 to May 30, 2021. These two countries have mainly experienced two waves of outbreaks, and the second outbreaks, when the number of confirmed cases and deaths increased rapidly, were more serious than the first wave. We assessed the effectiveness of the epidemic prevention measures taken by the two countries in combination with the epidemic situation.

### Data and sources

All epidemiological data, including cumulative confirmed cases, daily new cases, cumulative deaths and daily new deaths, were from the public data of the World Health Organization [12]. With regard to policy information concerning COVID-19, we searched national documents and responses to COVID-19 through government websites, such as the government decrees issued by the official website of the United States government (USAGov) and the official website of the India government (MyGov) since the outbreak of the epidemic. According to the collected epidemiological data and policy information from January 20, 2020 to May 30, 2021, we drew figures by Microsoft Excel for quantitative analysis.

## Results

### Main measures taken by the two countries in response to COVID-19

#### The United States

On January 20, 2020, the first confirmed case of COVID-19 appeared in the United States [12]. At the beginning of the epidemic, in the US, the government, the media, and the public were not aware of the COVID-19 epidemic, and the United States Centers for Disease Control and Prevention (CDC) advised the public to take daily precautions, but did not recommend that healthy people wear masks. With the increase of confirmed cases, the US government prepared to take isolation and curfew measures in "hot spots" on March 16. In late March, most US states and territories issued stay-at-home orders, stopped non-essential businesses and closed most schools. However, the states gradually reopened businesses and restaurants in late April and resumed work and production in May. While the epidemic did not completely slow down, the states’ epidemic prevention measures were relaxed. The number of daily new cases in the United States increased rapidly in late June and reached the peak of the epidemic on July 18 [12]. Subsequently, the United States took measures such as increasing investment in virus testing and advocating social distancing to alleviate the epidemic, but large-scale campaign rallies brought about by the presidential race, low public awareness of epidemic prevention, uneven epidemic prevention measures in various states, and the emergence of new coronavirus variants made the epidemic in the United States more severe. At the beginning of January 2021, the United States reached a new peak of more than 310,000 new cases per day [12]. The government has strengthened the implementation of epidemic prevention measures, such as strictly requiring individuals to wear masks in the transportation network, and strictly restricting restaurants, shops and other public places in the states. Since then, the number of daily new cases has decreased significantly, but the cumulative confirmed cases in the United States have been still the first in the world. Table 1 shows the main epidemic prevention and control policies adopted by the United States.

**Table 1:** The main epidemic prevention and control policies in the United States^a^

Policy	The key elements
1. Border prevention and control measures	(1) On February 2, 2020, non-US citizens from or recently in China were prohibited from entering. (2) On March 21, 2020, the federal government closed the US borders with Canada and Mexico. (3) On September 14, 2020, the government canceled the requirements for directing all flights carrying airline passengers arriving from, or recently had a presence in, certain countries to land at one of 15 designated airports and stopped strengthening entry health screening for these passengers. It also strengthened entry health screening for people arriving from, or with recent presence in, China (excluding Hong Kong and Macau), Iran, the Schengen region of Europe, the United Kingdom (excluding overseas territories outside of Europe), Ireland, and Brazil. (4) On December 28, 2020, the CDC asked air travelers from the United Kingdom to the United States to provide a negative test for the virus within 72 hours before boarding. (5) On January 26, 2021, the CDC required all air passengers entering the United States from foreign countries to be tested within 3 days before flight departure and to present an order certifying a negative test before boarding.
2. Measures for anti-epidemic materials	(1) On March 28, 2020, the first temporary hospital in New York was completed. (2) On August 23, 2020, the FDA approved the use of recovered plasma to treat patients with severe COVID-19. (3) On January 5, 2021, the FEMA revised the list of Personnel Protective Equipment and other scarce and critical health and medical resources that will be reviewed and may be reserved for domestic use before export, including surgical N95 respirators, nitrile gloves, exam gloves, etc.
3. Declaring a state of emergency across the country	(1) Starting from March 5, 2020, various states in the United States have entered a state of emergency. (2) On March 13, 2020, US President Trump declared a national emergency. (3) On April 1, 2020, US President Trump approved 30 states to enter the "disaster state" of the epidemic. (4) On April 11, 2020, US President Trump approved Wyoming as a "major disaster state" for the COVID-19 epidemic. It was the first time in the history of the United States that all 50 states, Washington D.C, and the four overseas territories including the U.S. Virgin Islands, Northern Mariana Islands, Guam and Puerto Rico have all entered a "major disaster state."
4. Testing and contact tracing measures	(1) On February 29, 2020, the FDA announced an "emergency use authorization" to expand testing capabilities. (2) On March 13, 2020, the FDA urgently approved a kit produced by the Swiss pharmaceutical company Roche, which can obtain test results within 3.5 hours and test 4128 samples within 24 hours. (0.12 total tests per thousand people) (3) On March 13, 2020, Trump stated that "drive-through" new coronavirus testing stations would be promoted nationwide in the coming weeks to improve testing capabilities. (4) Since early April 2020, teams appointed by state governors have worked with experts to develop state testing plans that include contact tracing testing and surveillance of asymptomatic individuals. (more than 5.07 total tests per thousand people) (4) On November 4, 2020, Dr. Robert Redfield, director of CDC, proposed the need for a new COVID-19 testing strategy to improve the ability to identify asymptomatic COVID-19 infections. (481.87 total tests per thousand people) (5) On February 17, 2021, the Biden government announced that it would take action to expand COVID-19 detection capabilities. The CDC would invest nearly $200 million to identify, track, and mitigate the emerging SARS-CoV-2 strains through genome sequencing. The HHS, in partnership with the DOD, would make a $650 million investment to expand testing opportunities for K-8 schools and underserved congregate settings, such as homeless shelters. (1000.21 total tests per thousand people) (6) Two new over-the-counter at-home COVID-19 tests were introduced to the US market in late December 2021. (more than 2095 total tests per thousand people)
5. Campus prevention and Control measures	(1) Starting from March 8, 2020, major colleges and universities across the United States suspended classes one after another. (2) Starting from March 12, 2020, primary and secondary schools in the United States from kindergarten to grade 12 of high school began to suspend classes for a period of time ranging from 2 to 6 weeks. (3) On July 6, 2020, the US Immigration and Customs Enforcement (ICE) announced that international students would be required to leave the country if all US schools began long-distance e-learning in the fall of 2020. At the same time, if the school only offered online courses, the immigration office would not issue visas to international students who were still abroad. (4) On July 12, 2020, the ICE said that the visas of American university students who were stranded outside the United States due to the outbreak and could only take online classes were still valid. (5) On July 14, 2020, the Trump administration officially lifted the rule that international students could not enter or stay in the United States with only online classes. (6) Schools in many states gradually reopened in early August 2020.
6. Maintaining social distancing and community control measures	(1) On March 14, 2020, the CDC had a No Sail Order in place, and the three major cruise companies, Carnival Group, Royal Caribbean, and Norwegian Cruise Line, have completely suspended cruise operations. (2) On March 16, 2020, the United States government considered taking measures such as quarantine and curfew in "hot spots", but not nationwide. (3) In late March 2020, people in the areas with more severe epidemics were required to stay at home. On March 30, Washington issued a stay-at-home order. (4) On May 13, 2020, Washington D.C Mayor Moore Bowser announced that the local state of emergency, public health emergency and "home order" would be extended to June 8. (5) On June 22, 2020, CDC released "Considerations for Election Polling Locations and Voters" making specific recommendations for reducing infectious behaviors, maintaining a healthy environment, and maintaining healthy practices. (6) On November 15, 2020, the Governor of Washington announced statewide restrictions, including prohibiting indoor social gatherings with people outside the family, closing restaurants and bars that provide indoor services, allowing take-out services, restricting outdoor dining, restricting the number of people in the religious service room, closing fitness facilities and gyms, etc. (7) On December 3, 2020, California, one of the "severely hit areas" of the epidemic, issued a stay-at-home order. (8) On February 2, 2021, the CDC required individuals to wear masks in all transportation networks in the United States, including airports, commercial aircraft, road buses, commuter buses, and rail systems, and then this requirement was extended to September 13.
7. Economic relief measures	(1) On March 18, 2020, the "Families First Coronavirus Response Act" was approved to provide two weeks of paid sick leave and family leave and increase government funding for health care, food benefits, and unemployment benefits. (2) On March 27, 2020, the "Coronavirus Aid, Relief, and Economic Security Act" was passed, providing $2.2 trillion in funding for individuals, small businesses, large corporations, state and local governments, and other public health initiatives, and it was also a major source of funding for medical interventions like vaccines and rapid testing. (3) In March 2021, more than $170 billion in new resources was allocated to the US Department of Education through the "American Relief Plan Act" to support ongoing states and agencies recovery efforts.
8. Vaccination measures	(1) On December 8, 2020, the government issued an executive order on securing access to the US government's COVID-19 vaccine. (2) On January 26, 2021, the government increased the weekly vaccine supply to states and territories from 8.6 million doses to at least 10 million doses, and planned to purchase an additional 200 million doses of vaccine to vaccinate people. (3) On May 10, 2021, the FDA extended the emergency use authorization of the Pfizer-BioNTech COVID-19 vaccine to teenagers between 12 and 15 years old.
9. Lifting restrictions	(1) From April 20, 2020, South Carolina and other states or regions gradually reopened businesses and restaurants. By the end of May, most states or regions opened businesses and restaurants. (2) In May, the United States began to see a concentrated "resumption of work and production", and many states introduced plans to restart the economy one after another. (3) The CDC announced that the "Navigation ban" would be lifted from November 2020 and replaced by the "Conditional Sailing Order". (4) On May 13, 2021, the CDC announced that in most cases, fully vaccinated Americans no longer need to wear masks indoors and maintain social distance. (5) On May 15, 2021, the CDC stated that many K-12 schools that had strictly implemented prevention strategies had been able to safely open for face-to-face instruction and remain open, and it recommended that schools implemented phased prevention strategies.

*Abbreviations:* CDC, the United States Centers for Disease Control and Prevention; FDA, the United

States Food and Drug Administration; ICE, the US Immigration and Customs Enforcement; HHS, the US Department of Health & Human Services

^a^ Table 1 is compiled from the policies and regulations published on the official websites of the United States government, the United States Centers for Disease Control and Prevention, the United States Department of Health & Human Services, the United States Food and Drug Administration and World Health Organization

#### India

On January 30, 2020, India confirmed its first case of COVID-19 infection [12]. India implemented a nationwide blockade policy on March 25, 2020 [13]. As the epidemic situation became more severe, India entered the first period of rapid deterioration of the epidemic in early July and reached a peak in September. To reduce the speed of the epidemic, the Indian government has adopted measures such as implementing border prevention and control measures, managing different regions according to the severity of the epidemic, strictly controlling the export of epidemic prevention medical resources, strengthening case tracking, phased blockades, etc. The epidemic situation has slowed down and restrictions have been lifted in stages. It is worth noting that the contact tracing strategy adopted by the United States is limited—not done for all cases, in contrast, India adopted a comprehensive contact tracing strategy in late September, which has played a positive role in its epidemic control [10]. However, the epidemic rebounded due to lax epidemic prevention measures, the mutation of the novel coronavirus, the dilution of public awareness of epidemic prevention and so on, which was similar to the situation in the United States [14]. In April 2021, the daily new cases increased rapidly, and the epidemic in India reached a new peak in early May [12]. Later, according to the actual situation, several states imposed curfews and restricted the opening of public places. Table 2 shows the main epidemic prevention and control policies adopted by India.

**Table 2:** The main epidemic prevention and control policies in India^b^

Policy	The key elements
1. Border prevention and control measures	(1) On January 25, 2020, the government issued a travel warning, requiring the public to avoid unnecessary trips to China. (2) On 2 February 2020, e-visa services to India for Chinese passport holders and visa applicants of other nationalities residing in China were suspended. (3) On March 11, 2020, the border was closed and passengers from countries with severe epidemics would be quarantined for 14 days after entering India. (4) From March 22, 2020, the border prevention and control policy was upgraded to prohibit the entry of international passengers.
2. Maintaining social distancing and community control measures	(1) Conduct community quarantine on close contacts. (2) The government recommended that state governments required their factories, shops and other entities to adopt a "Work From Home Policy" and pay wages as usual. (3) Keep a distance of at least 6 feet in public places.
3. The blockade measures	(1) The "Janta" curfew was implemented on March 22, 2020, from 7 a.m. to 9 p.m., prohibiting anyone from leaving their houses except people providing basic services. (2) From March 25, 2020, the whole country entered a 21-day full lockdown state, which was then extended until 31 May. Residents, except those with necessary occupations, were required to stay at home. During the lockdown, all shops, commercial establishments, factories, workshops, offices, markets and places of worship would be closed, and interstate buses, subways and construction activities would also be suspended. (3) On May 4, 2020, the states were divided into three zones: red, orange and green, and strict "stay-at-home orders" should be implemented in the red and orange zones, while relevant activities were allowed in the green zone. The order was valid until May 17. (4) On 17 May 2020, the red and orange zones were divided into containment zones and buffer zones. No one was allowed to enter or leave the containment zone and only necessary activities were allowed within the containment zone. The order was valid until May 30. (5) On April 13, 2021, Maharashtra announced that it would implement a "national curfew" from 8 pm on the 14th to 7 am on May 1. (6) On April 19, 2021, New Delhi implemented a city-wide blockade, and announced the extension of the blockade four times on April 25, May 1, May 9, and May 16. (7) In May 2021, curfews were imposed in many places and Madhya Pradesh was completely sealed off.
4. Campus prevention and control measures	(1) Allow and encourage online or distance learning. (2) Close all educational institutions (including schools and universities) from 16 March 2020. (3) On September 21, 2020, in areas outside the quarantine area, up to 50% of teaching and non-teaching staff could be allowed to go to the school for online teaching or remote tutoring. (4) The Ministry of Education urged the postponement of offline examinations in May 2021.
5. Medical resource measures	(1) The export of protective equipment such as masks and protective clothing was prohibited from January 31, 2020. (2) India transformed 20,000 train carriages into isolation wards, which can accommodate up to 320,000 beds that meet the isolation requirements. (3) On April 12, 2020, major hospitals have prepared 106,000 beds to receive patients whose number may surge at any time. (4) On March 25, 2021, the export of vaccines was restricted.
6. Testing and contact tracing measures	(1) On April 2, 2020, the "ArogyaSetu" mobile app for digital contact tracing was launched in India. (0.03 total tests per thousand people) (2) The ALLA began to provide free testing and treatment facilities for COVID-19 patients on 30 July 2020. (13.5 total tests per thousand people) (3) Conduct close contact tracing and door-to-door testing in the quarantine area according to actual needs. (4) It was announced that to improve detection capacity, RT-PCR detection should be expanded to 70% or more as far as possible from April 1, 2021. (5) In late December 2021, the Indian government asked states in writing to promote at-home testing, especially for those showing symptoms of infection. (more than 471 total tests per thousand people)
7. Vaccination measures	The vaccination program began on 16 January 2021.
8. Lifting restrictions in stages	(1) On April 20, 2020, the opening of agricultural enterprises, including dairy products, aquaculture and plantations, was allowed. Cargo transportation vehicles were allowed to operate. Banks and government centers that distribute benefits were also allowed to open. (2) Gradually restart passenger train operations from May 12, 2020. (3) On May 30, 2020, the government announced the lifting of the blockade restrictions outside the quarantine zone. The first phase of the closure began on June 1, 2020, and the curfew time was shortened. The Government gradually lifted the ban and allowed some enterprises to resume work and production. (4) On July 1, 2020, the second phase of unsealing began, and the curfew time was shortened. (5) On August 1, 2020, the third phase of unsealing began. (6) On September 1, 2020, the fourth phase of unsealing began, allowing further opening of public places under certain restrictions. (7) From October 15, 2020, the fifth phase of unsealing began, which allowed cinemas and theatres outside the blockaded areas to hold up to 50% of the seats, and allowed the opening of entertainment parks and other similar venues. (8) On 22 October 2020, the government gradually eased visa and travel restrictions and allowed all OCI and PIO cardholders and all other foreign nationals (except those on tourist visas) planning to visit India for any purpose to enter India by air or by water through approved immigration checkpoints at airports and seaports. (9) On 27 January 2021, new surveillance, containment and warning guidelines were issued, providing that all activities were permitted except a few that were permitted under certain restrictions. The phased development, gradual resumption, etc., would continue.

^b^Table 2 is compiled from the policies and regulations published on the official websites of the India government, Ministry of Health and Family Welfare of India and World Health Organization

### The effect of the epidemic prevention measures taken by the two countries

According to the trend of the epidemic curve in the United States and India, the two countries have roughly experienced two epidemic stages, that is, the epidemic in the United States can be divided into two stages on September 9, 2020, and the epidemic in India can be divided into two stages on February 2, 2021. However, there are differences in the epidemic curves of the two countries. The two epidemic peaks of India are clearly demarcated, and there is an effective decline in the epidemic situation between the two peaks [15]. But the boundary between the two peaks of the epidemic in the United States is blurred. Moreover, it can be clearly seen from the figure that in the first wave of the epidemic in the United States, the epidemic curve fluctuated repeatedly and the curve fell incompletely.

#### The United States

**Fig. 1:**
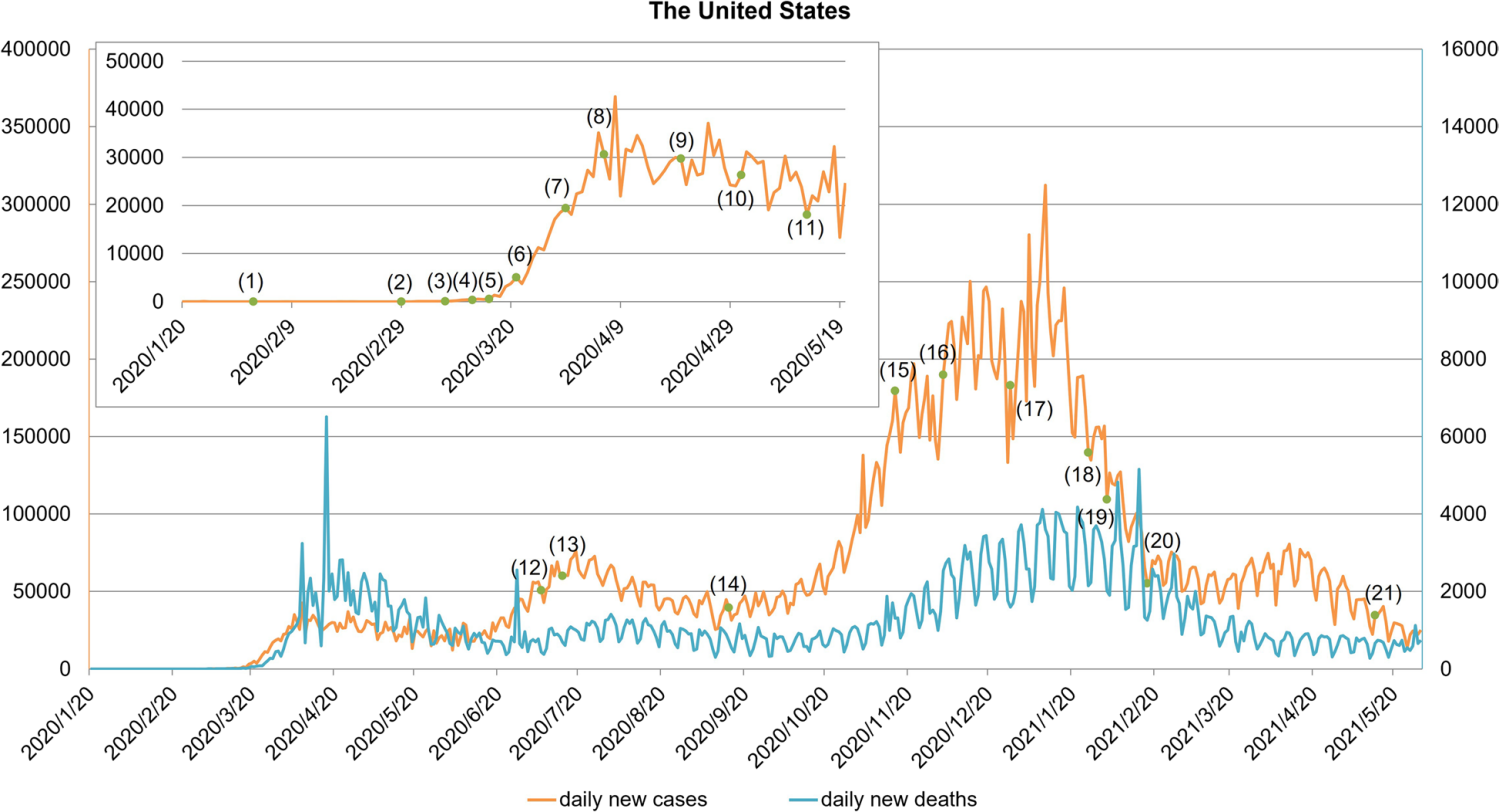
The COVID-19 epidemic curve of the United States. Notes: (1) On February 2, 2020, non-US citizens from or recently in China were prohibited from entering. (2) On February 29, 2020, the FDA announced an "emergency use authorization" to expand testing capabilities. (3) From March 8, 2020, the colleges and universities across the United States suspended classes one after another. (4) On March 13, 2020, US President Trump declared a national emergency. (5) On March 16, 2020, the government considered taking measures such as quarantine and curfew in "hot spots", but not nationwide. (6) On March 21, 2020, the federal government closed the US borders with Canada and Mexico. (7) On March 30, 2020, Washington issued a stay-at-home order. (8) On April 6, 2020, the CDC announced that it would conduct antibody tests on a wider range of people to determine whether more people had been infected with COVID-19 and monitor the immunity of patients after they had recovered. (9) From April 20, 2020, South Carolina and other states or regions gradually reopened businesses and restaurants. (10) On May 1, 2020, more than 20 states, including Texas and Georgia, entered the stage of resuming work and production, and many states have successively introduced plans to restart the economy. (11) On May 13, 2020, Washington D.C Mayor announced extending the local state of emergency, public health emergency and the implementing of the "home order". (12) On July 6, 2020, the ICE announced that international students would be required to leave the country if all US schools began long-distance e-learning in the fall of 2020. And if the school only offered online courses, the immigration office would not issue visas to international students who were still abroad. (13) On July 14, 2020, the rule that international students could not enter or stay in the United States with only online classes was officially lifted. (14) From September 14, 2020, the USG canceled the requirements for directing all flights carrying airline passengers arriving from, or recently had a presence in, certain countries to land at one of 15 designated airports and stopped strengthening entry health screening for these passengers. (15) On November 15, 2020, the Governor of Washington announced statewide restrictions. (16) On December 3, 2020, California issued a stay-at-home order. (17) On December 28, 2020, the CDC required air passengers from the UK to the US to provide evidence of a negative virus test within 72 h before boarding. (18) On January 26, 2021, the CDC expanded the COVID-19 negative test requirements for all air passengers entering the US, requiring passengers arriving in the US from foreign countries to be tested within 3 days before flight departure and to present an order certifying a negative test before boarding. (19) On February 2, 2021, the CDC required individuals to wear masks in all transportation networks in the United States. (20) On February 17, 2021, the Biden government announced the upcoming action on expanding COVID-19 testing capabilities. (21) On May 13, 2021, the CDC announced that in most cases, fully vaccinated Americans no longer need to wear masks indoors and no longer need to maintain social distance

In the initial period of the outbreak in the United States, the United States mainly banned non-US citizens from or recently in China. Internally, the CDC advised the public to take daily precautions which did not include requiring healthy people to wear masks. And the US government believed that the COVID-19 virus was similar to ordinary flu, which to a certain extent misled people about the epidemic. The awareness of epidemic prevention of the US government departments and the American public was not high [16]. In March 2020, the epidemic situation in the United States showed a severe trend. On March 13, the country declared a state of emergency [17]. On March 27, the United States surpassed the data released by China and Italy and became the country with the worst epidemic [12]. The FDA gave emergency approval to a virus test kit made by Swiss pharmaceutical company Roche to improve the efficiency of case detection. As testing has increased, the severity of the outbreak has also increased, although the United States surpassed 10 total COVID-19 tests per 1,000 people in early April, surpassing South Korea, where the outbreak was better controlled at that time [10, 18]. The states entered a state of emergency, issued home orders, and restricted the opening of public places. And then the epidemic was alleviated to a certain extent. But soon after, states’ loosened control measures, the spread of a mutant strain, large-scale presidential campaign rallies, and inadequate public awareness of the SARS-CoV-2 worsened the epidemic in the United States, with the daily number of new confirmed cases rising rapidly and peaking on January 10, 2021. Many states entered a new round of restrictions and the "mask order" was more strictly enforced. The outbreak was rapidly alleviated in February. Subsequently, the states’ epidemic prevention measures were gradually relaxed, and fully vaccinated Americans no longer need to wear masks indoors and maintain social distance in most cases. Although the epidemic has eased to some extent, by May 30, 2021, the United States had more than 32.91 million cumulative confirmed cases and more than 580,000 cumulative deaths, both ranking first in the world. Figure 1 is the COVID-19 epidemic curve of the United States.

#### India

**Fig. 2:**
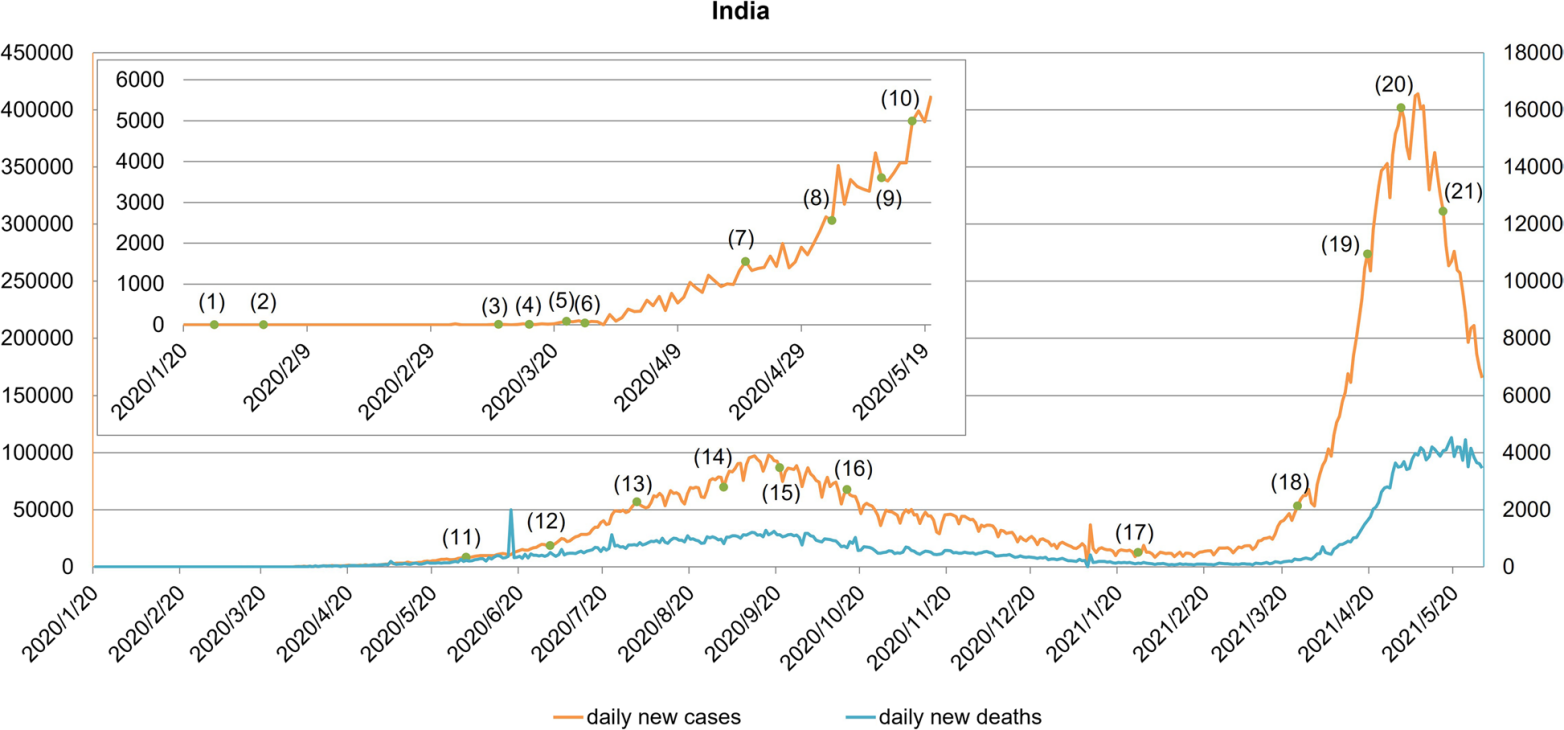
The COVID-19 epidemic curve of India. Notes: (1) On January 25, 2020, the government issued a travel warning, requiring the public to avoid unnecessary trips to China. (2) On February 2, 2020, the Indian electronic visa service for Chinese passport holders and visa applicants of other nationalities residing in China was suspended. (3) On March 11, 2020, the border will be closed, and passengers from countries with severe epidemics would be quarantined for 14 days after entering the country. (4) From March 16, 2020, the schools and universities were closed. (5) On March 22, 2020, the "Janta" curfew was implemented. (6) On March 25, 2020, India began a 21-day complete lockdown. (7) On April 20, 2020, the opening of agricultural enterprises, including dairy products, aquaculture and plantations, was allowed. Cargo transportation vehicles were allowed to operate. Banks and government centers that distribute benefits were also allowed to open. (8) On May 4, 2020, the government divided the states into red, orange, and green areas for district management. (9) From May 12, 2020, passenger train operations were gradually restarted. (10) On 17 May 2020, the red and orange zones were divided into containment zones and buffer zones. People in the containment zone were not allowed to enter and exit, and only necessary activities were allowed. (11) The first phase of unblocking began on June 1, 2020. (12) On July 1, 2020, the second phase of unsealing began. (13) On August 1, 2020, the third phase of unsealing began. (14) On September 1, 2020, the fourth phase of unsealing began. (15) On September 21, 2020, in areas outside the quarantine area, up to 50% of teaching and non-teaching staff could be allowed to go to the school for online teaching or remote tutoring. (16) On October 15, 2020, the fifth phase of unsealing began. (17) On 27 January 2021, new surveillance, containment and warning guidelines were issued, providing that all activities were permitted except a few that were permitted under certain restrictions, and phased development, gradual resumption, etc., would continue. (18) On March 25, 2021, India restricted the export of vaccines. (19) On April 19, 2021, New Delhi implemented a city blockade. (20) In May 2021, curfew measures were implemented in many places, and Madhya Pradesh was completely blocked. (21) On May 16, 2021, New Delhi announced the fourth extension of the blockade

India's focus in the initial phase of the outbreak was mainly on border control, such as the January 25, 2020 travel warning which asked the public to avoid non-essential travel to China. After the first confirmed case of COVID-19 appeared, India strictly controlled epidemic prevention materials and quarantined close contacts in the community. The number of confirmed cases of COVID-19 has stabilized at a low level. In early March, India mainly dealt with imported cases from Europe, the Middle East, Japan, South Korea and other countries, and local cross-infections gradually appeared. Particularly, the tour groups from Italy caused a sudden increase in the number of COVID-19 cases in India [19]. In the face of the epidemic, the Indian government closed the border, and Prime Minister Modi advocated the implementation of a "Janta" curfew which prepared for the complete blockade for the next 21 days. On March 25th, curfew measures were adopted, relevant public places were closed, and the three colors were used to divide states and regions to implement different management strategies. The epidemic situation has changed slowly. In June, India began lifting restrictions in stages, with the resumption of work and production taking place gradually, at the same time, the daily new cases increased rapidly. After the fourth phase of the lifting, the severity of the epidemic in India peaked, and then gradually eased. At the end of March 2021, the situation of the epidemic in India deteriorated sharply, coupled with the lack of medical resources, the epidemic in India reached a peak on May 6, with the number of confirmed cases exceeding five times the first peak of the outbreak [12, 14]. As a result, the government restricted the export of vaccines and imposed a new round of lockdown and curfews measures in several states. The daily new cases have shown a downward trend, but the daily new deaths have not shown a significant downward trend. India's epidemic situation remained critical, with more than 27.89 million cumulative confirmed cases and more than 320,000 cumulative deaths as of 30 May 2021. Figure 2 is the COVID-19 epidemic curve of India.

## Discussion

While many vaccines have been developed and successfully put into use now, non-pharmaceutical interventions are also important, and the combination of the two can accelerate the success of the global fight against COVID-19. In the COVID-19 pandemic, countries have adopted different interventions at different stages according to their specific national conditions, with different implementation effects. This study described the intervention strategies and specific measures, which were adopted in the United States and India in response to the COVID-19 outbreak, and evaluated the effectiveness of the policies based on the daily number of new cases and deaths in each country.

In terms of the policies adopted by the United States and India in this epidemic, the United States has adopted a mitigation strategy. Its prevention and control goals were to control the spread, delay the epidemic speed, and reduce the overall harm. The focus of the implementation was on the treatment of severely ill patients [20], reflecting the characteristic of "treatment over prevention". India has adopted a combination of containment and mitigation. Unlike mitigation, containment aims to completely stop the spread of the virus, preferring more aggressive closed management measures.

### The similarities between the two countries in fighting the epidemic

#### Adopting mitigation strategies

The United States has generally adopted a mitigation strategy. An obvious example is that after the outbreak worsened dramatically in the second half of 2020, the president of the United States called for Americans to wear masks and states promulgated measures to limit social distance. On November 15, the Governor of Washington announced statewide restrictions, including prohibiting indoor social gatherings with people outside the home, closing gyms, restaurants and bars that provide indoor services, limiting the number of religious services to 25% of indoor capacity or 200 people, and so on. On December 3, California, where the epidemic was serious, issued a stay-at-home order. After January 2021, the epidemic in the United States has shown a downward trend, but it has not yet reached the time when the policy can be completely relaxed. India adopted a comprehensive blockade containment strategy in the early stage of the epidemic, but it has brought serious economic impact [21]. Although the government has issued an economic stimulus plan, experts said its actual stimulus effect was relatively poor. After the epidemic escalated, India no longer implemented a nationwide lockdown strategy and it was only that some states took some restrictive measures based on the actual situation. Various data showed that the epidemic situation in India was still severe.

#### Premature easing of anti-epidemic policies

In the face of the epidemic, after the United States declared a state of emergency, stay-at-home orders were issued in many states in late March 2020. However, by the end of April, when there was no clear indication that the epidemic had been successfully controlled, states or territories began to ease the requirements of social distance, and implement the order of reopening, which is likely to be a political response to the high unemployment rate caused by the government shutdown, rather than a public health response to COVID-19 infection [22]. In May, there was a concentrated "resumption of work and production", coupled with the intensification of social conflicts such as the Freudian incident bringing large-scale crowds [23], which further promoted the escalation of the COVID-19 epidemic in the United States. It can be seen that it is necessary to be vigilant when relaxing epidemic prevention measures, and it is very necessary to maintain control measures before achieving reasonable public health objectives [24].

Similarly, India implemented a total lockdown policy on 25 March 2020, but before the epidemic stabilized and the daily new cases showed a significant decline, India implemented a phased unblocking strategy from June, with the first wave peaking in mid-September. Since then, virus mutation, large-scale political rallies and religious events, and lack of medical resources have contributed to the rapid deterioration of the epidemic in India. It can be seen that premature relaxation of the policy will bring the risk of repeated epidemics. As of May 30, the two countries have shown signs of easing the epidemic, but if they loosen policy too soon, they may repeat the same mistake. In India in particular, daily new cases only declined slightly and daily new deaths remained high, but there were signs that the Indian government began gradually easing restrictions in June, allowing factories to operate and building activities to resume gradually [25], which may lead to a rebound of the epidemic in India.

### The differences between the two countries in fighting the epidemic

In the fight against the epidemic, the United States focused on treating patients with severe cases, encouraged those with mild symptoms to stay at home, and avoided large-scale lockdown strategies even when the outbreak deteriorated sharply. The cumulative number of COVID-19 cases in the United States now ranks first in the world. And it should be added that under the national system of the United States, the federal government left most of the decision-making power to deal with COVID-19 to the States [26], which contributed to the impossibility of the large-scale blockade strategy to a great extent. India, by contrast, in the initial stage of the epidemic, focused its decision-making on the national level, adopted a strict containment strategy, implemented a 21-day lockdown strategy across the country, and through the national surveillance network "Comprehensive Disease Surveillance Plan" deployed hundreds of public health workers to monitor millions of citizens in rural, suburban, and urban communities and detect disease clusters early, which to a certain extent contributed to India’s effectiveness in controlling the spread of the virus in the early stages of the epidemic [27].

In terms of implementing containment strategies, Chinese practice is a typical case. China through strict containment measures such as timely lockdown of Wuhan, strict management of the "four types of people" (confirmed cases, presumptive cases, fever cases, and close contacts) and so on, has successfully brought the epidemic under control without large-scale recurrence of the epidemic. From this point of view, compared with mitigation strategies, containment strategies can control the epidemic more quickly to a certain extent.

In general, in the early stage of the epidemic, India adopted containment strategies, but after that, both the United States and India adopted mitigation strategies. At present, despite the continuing threat of the mutant strain of the virus, there are signs of lax national epidemic prevention policies. For example, by February 2022, many states in the United States have abolished mask laws. Of course, vaccination efforts around the world are in full swing, including the Indian government's expansion of vaccination centers and increased funding in the United States to expand community-based outreach efforts to increase vaccination [28]. At the same time, however, vaccination efforts are also challenged by many factors such as vaccination hesitancy and unequal global distribution of vaccines, and the acceptance and uptake of vaccines are not ideal [11, 29]. WHO experts said that vaccines alone would not end the pandemic, stressing the need to continue to take measures that have been shown to slow the spread of the virus [30]. In the face of the still severe domestic epidemic, the United States and India should take appropriate epidemic prevention measures while accelerating the implementation of vaccination. And according to specific national conditions, containment strategies for faster control of the epidemic can be considered.

## Conclusion

There are differences between the epidemic prevention strategies adopted by the United States and India, especially India’s containment strategies which helped it better control the epidemic in the early stage. However, the epidemic in the two countries is still severe in the world. When the virus mutants are coming and the immune barrier is not formed, it is meaningful that the two countries continue to take non-pharmacotherapy intervention measures and accelerate vaccination, according to specific national conditions adopt containment strategies that can control the epidemic more quickly when necessary, and pay attention to the risk of epidemic rebound caused by premature relaxation of epidemic prevention policies.

## Data Availability

All data generated or analysed during this study are included in this published article.
